# Robust Uptake of Magnetic Nanoparticles (MNPs) by Central Nervous System (CNS) Microglia: Implications for Particle Uptake in Mixed Neural Cell Populations

**DOI:** 10.3390/ijms11030967

**Published:** 2010-03-08

**Authors:** Mark R. Pickard, Divya M. Chari

**Affiliations:** Cellular and Neural Engineering Group, Institute for Science and Technology in Medicine, Keele University, Staffordshire, ST5 5BG, UK; E-Mail: m.r.pickard@biol.keele.ac.uk

**Keywords:** magnetic, nanoparticles, microglia, toxicity, uptake

## Abstract

Magnetic nanoparticles (MNPs) are important contrast agents used to monitor a range of neuropathological processes; microglial cells significantly contribute to MNP uptake in sites of pathology. Microglial activation occurs following most CNS pathologies but it is not known if such activation alters MNP uptake, intracellular processing and toxicity. We assessed these parameters in microglial cultures with and without experimental ‘activation’. Microglia showed rapid and extensive MNP uptake under basal conditions with no changes found following activation; significant microglial toxicity was observed at higher particle concentrations. Based on our findings, we suggest that avid MNP uptake by endogenous CNS microglia could significantly limit uptake by other cellular subtypes in mixed neural cell populations.

## 1. Introduction

Microglia are a major class of glial cells (the non-neuronal, support cells of the brain) that constitute the macrophages of the central nervous system (CNS); these cells are widely believed to be of haematopoetic origin and populate the CNS during development (reviewed in [1–3]). These are a ubiquitous cell population distributed throughout the CNS and constitute approximately 10% of the total glial cell population [3], a number thought to approximate that of neurons. Microglial processes show high motility and, even under so-called ‘resting’ conditions, constantly survey the local microenvironment for endocytosis of nutrients and clearance of cellular debris [2,3]; microglia possess the full range of endocytotic mechanisms, including receptor-mediated endocytosis, macropinocytosis and phagocytosis [4–7]. Indeed, microglia are highly phagocytic cells; they constitute the first line of defence against invading pathogens and are additionally involved in the recruitment of immune cells from the periphery into areas of pathology [1–3,8]. Given the dual role of microglia as immune effector cells and in the ‘trafficking’ of immune cells into the CNS, it is increasingly believed that therapeutic manipulation of the functional properties of microglia could constitute an important strategy to promote neural repair [2,8].

In the normal adult CNS, microglia display highly ramified morphologies, but these cells can demonstrate considerable morphological plasticity under pathological conditions [1]. The process of microglial ‘activation’ (as occurs in a range of neuropathological situations including infectious disease, neural injury and neurodegenerative conditions) causes these cells to assume hypertrophic, rounded, amoeboid morphologies that enable them to undertake their macrophage activities [1]. In this activated state, microglia also rapidly upregulate the expression of several receptors and secrete a range of products, many implicated in the pathogenesis of neuroinflammatory and neurodegenerative conditions [1–3,8].

Magnetic nanoparticles (MNPs) are a major new class of contrast agent that have been used to image the roles of microglia/macrophages in foci of neural injury and disease and as such, have been the focus of considerable attention in recent years [9–13]. MNPs have been proven to be a valuable tool in the detection of neuroinflammatory foci [13], delineation of tumours [9,13] and nerve injury [10] and are useful agents for monitoring neuropathological processes such as macrophage infiltration and disruption of the blood brain barrier (BBB) [10,13]. In histological assessments of such areas of pathology, reactive microglia/macrophages have been shown to take up MNPs and ‘trap’ particles in trauma sites [9–11,13]. While MNPs have major imaging applications, it is also important to note that such particles constitute novel and important vector systems for the noninvasive, magnetic targeting of drugs and biomolecules (including siRNA, DNA, shRNA) in a range of physiological systems [14–16]. This diversity and flexibility in MNP applications (biomolecule delivery, magnetic targeting and imaging) could therefore allow such particles to form the basis of a ‘multifunctional nanoplatoform’ for neural regeneration applications involving microglial cells. However, in order to establish the feasibility of such an approach, it will be critical to assess the extent and time course of uptake of MNPs by microglia, their subsequent intracellular processing and potential toxic effects associated with such particles. In this context, some studies have documented the time course of uptake of MNPs by primary microglial cells [9,17], but it is not clear if the process of microglial activation *per se* can alter MNP uptake and intracellular processing by these cells. This is clearly an important question to address when considering strategies to promote neural regeneration, as microglial activation occurs following almost all CNS pathologies [1–3].

To address these issues, we have studied MNP uptake in microglia in both basal medium and after treatment with the activator lipopolysaccharide (LPS) [18]. Additionally, induction of proliferation is one of the first steps in microglial activation (and is key to the induction of CNS inflammation) [19]. Therefore, in order to distinguish alterations in particle uptake due to microglial activation from those due to mitogenic effects, cells were also treated with a mitogenic (but non-activating) dose of granulocyte/macrophage colony-stimulating factor (GM-CSF) [20]. The following parameters were assessed (i) the time course and dose-dependence of MNP uptake; (ii) the intracellular fate of the particles; (iii) the effects of experimental stimulation of microglial cells (proliferation and activation) on MNP uptake and intracellular processing and; (iv) toxicity associated with MNP uptake under basal and stimulated conditions.

## 2. Results

### 2.1. Particle and Culture Characteristics

**Figure 1.**
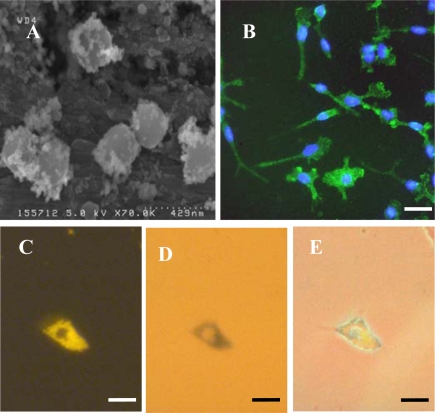
MNP uptake by microglial cultures: particle and culture characterization. **(A)** SEM image of Spherofluor MNPs demonstrating uniformity of size of particles. **(B)** Double-merged image of microglia immunostained with OX-42 antibody (green) and mounted with medium containing the nuclear marker, DAPI (blue) demonstrating high culture purity. **(C)** Nile Red fluorescence associated with MNPs in microglial cell at 24 h after labelling with 20 μg/mL MNPs for 1 h. **(D)** Light micrograph of cell depicted in (C) showing blue deposits of iron-containing material detected by Perl’s staining. **(E)** Merge of images C & D demonstrating co-localization of iron and Nile Red fluorescence, reflecting the stability of MNPs in microglia. Scale bar in (B) = 50 μm and in (C–E) = 25 μm.

**Figure 2.**
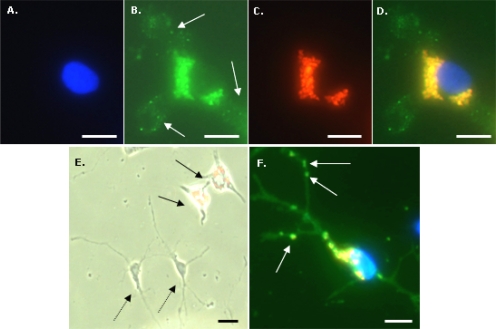
Avid particle uptake by microglial cells. **(A)** DAPI fluorescence of microglial cell incubated with 2 μg/mL MNP for 24 h. **(B)** OX-42 fluorescence of cell depicted in (A); arrows indicate microglial processes. **(C)** Nile Red fluorescence associated with MNPs in cell depicted in (A). **(D)** Triple-merge of images depicted in (A–C) demonstrating accumulation of large collections of particles by microglia with a striking perinuclear localisation **(E)** Double-merged image of Nile Red fluorescence (MNPs; red) and phase-contrast micrograph of cells 7 days after pulse-labelling with 20 μg/mL MNPs demonstrating particle uptake in microglia (solid arrows) but not in neighbouring cells which morphologically resemble OPCs (dashed arrows). **(F)** Triple-merged image of DAPI (blue), A2B5 (green) and Nile Red (MNPs; red) associated fluorescence demonstrating accumulation of MNPs by purified OPC cultures after incubation with 20 μg/mL MNPs for 24 h. Note the presence of MNPs in processes (arrows) in addition to perinuclear/cytoplasmic localization. Scale bar = 10 μm in (A–D) & (F), and 50 μm in (E).

Analysis of Spherofluor MNPs by SEM revealed that these were of regular shape and size (Figure 1A), consistent with the size range (200–390 nm diameter; mean = 360 nm) reported by the manufacturer. Microglial cultures of high purity (96.8 ± 0.5%; n = 5) were reliably obtained in this study, as judged by immunostaining with the OX-42 antibody (Figure 1B); the majority of contaminating (OX-42 negative) cells were morphologically distinct from microglia and resembled oligodendrocyte precursor cells (OPCs) (Figure 2E). Confocal microscopic analysis revealed that fluorescence associated with DAPI, OX-42 and MNPs was coincident; Nile Red fluorescence was only clearly visible in internal slices of cells. Cell-associated fluorescence was therefore inferred to represent internalized MNPs. Perl’s staining of microglia which had been pulse labelled with MNPs for 1 h, then cultured in the absence of particles for 24 h, revealed co-localization of iron-containing material (blue deposits) and Nile Red fluorescence (Figure 1C–E), suggesting that MNPs remained chemically stable *post*-ingestion.

### 2.2. Uptake and Intracellular Processing of Spherofluor MNPs

Microglia showed rapid and avid uptake of MNPs (Figure 2A–D); interestingly, whilst extensive accumulation of Spherofluor MNPs was clearly demonstrable in microglia, it was rarely seen in adjacent (OX-42 negative) cells that morphologically resembled OPCs (Figure 2E). In striking contrast, purified cultures of OPCs (96.3 ± 0.6% A2B5-positive cells) that had been exposed to an identical concentration range of MNPs showed avid particle uptake (Figure 2F).

As MNP concentration and incubation time were increased, particles showed a striking tendency to ‘stream’ towards the nucleus and consequently, with time, showed distinctive aggregations in the cytoplasm. Particles that demonstrated such a tendency to form aggregates around the nucleus were classified as perinuclear (Figure 2D).

Microglial uptake of MNPs was temperature-dependent. Incubation of cells with 20 μg/mL MNPs for 1 h at 4 °C, resulted in a marked reduction (by 78%) in the proportion of cells showing uptake when compared with parallel incubations conducted at 37 °C; the vast majority (95%) of cells labeled at 4 °C showed low levels of particle accumulation (data not shown).

**Figure 3.**
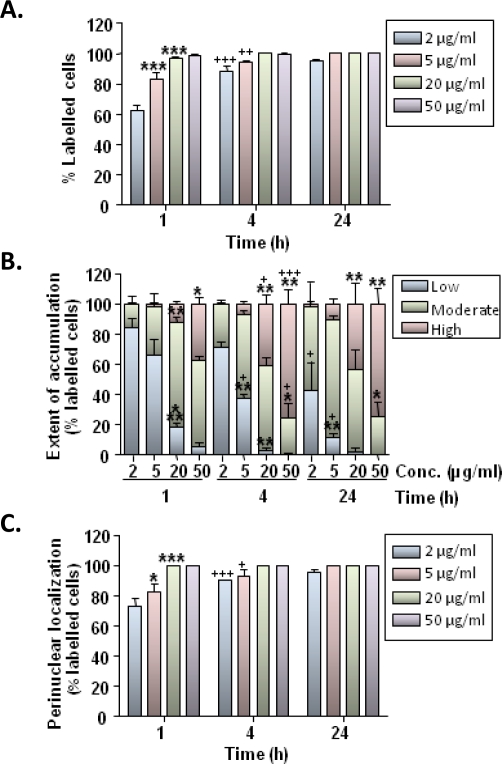
Time course and concentration-dependence of MNP uptake in microglia. **(A)** Bar chart showing the proportion of OX-42-positive cells showing MNP uptake. **(B)** Stacked bar chart showing the extent of particle accumulation by cells. **(C)** Bar chart showing proportion of labelled cells with a perinuclear particle localization (either exclusively or combined with a cytoplasmic distribution). *P < 0.05, **P < 0.01 and ***P < 0.001 *versus* preceding MNP particle concentration at the same time point; ^+^*P* < 0.05, ^++^*P* < 0.01 and ^+++^*P* < 0.001 *versus* same concentration at preceding time point (two-way ANOVA and Bonferroni’s post-tests; n = 3 cultures).

Microglial uptake of MNPs was also both time- and concentration-dependent (Figure 3). After 1 h of particle application, the majority of cells showed labelling with particles (Figure 3A). This ranged from a mean value of 63% for incubations containing 2 μg/mL MNPs to 99% for incubations containing 50 μg/mL MNPs (Figure 3A). By 4 h, the proportion of cells labelled with 2 μg/mL MNPs had increased to 89%, whilst labelling of nearly all cells was apparent in incubations containing ≥20 μg/mL MNPs (Figure 3A). By 24 h, 95% of cells incubated with 2 μg/mL MNPs showed particle uptake, whilst labelling of all cells was seen at higher MNP concentrations (Figure 3A).

The extent of intracellular MNP accumulation by microglia also increased with particle concentration and incubation time (Figure 3B). The proportion of cells displaying moderate-to-high levels of particle accumulation gradually increased between 1 and 24 h in incubations containing 2 and 5 μg/mL MNPs, whilst this parameter was maximal at 4 h in incubations containing 20 and 50 μg/mL MNPs (Figure 3B). Nevertheless, at 24 h, high levels of particle accumulation were seen in only a minor proportion of microglia incubated with 2 and 5 μg/mL MNP, whilst 43 and 75% microglia were characterized as showing high levels of MNP accumulation at 20 and 50 μg/mL MNP, respectively (Figure 3B).

In incubations with 2 μg/mL MNPs for 1 h, the proportion of labeled microglia displaying particles in a perinuclear localization (either exclusively or in combination with cytoplasmically localized particles) was 73%, whereas this proportion had increased to 91% at 4 h (Figure 3C). The majority (>90%) of labelled microglia also showed particle accumulation in the perinuclear region by 4 h for incubations with 5 μg/mL MNPs, whereas a 1 h incubation was sufficient to achieve this for cells treated with ≥20 μg/mL MNPs (Figure 3C).

### 2.3. Effect of Microglial Stimulation on Particle Uptake

**Figure 4.**
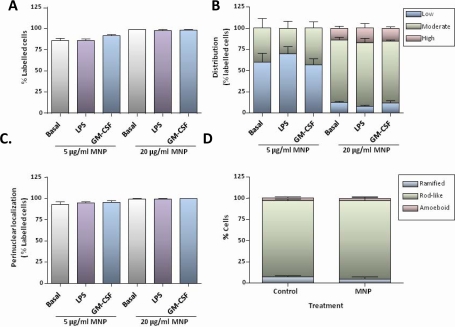
Effect of microglial stimulators on MNP uptake. **(A)** Bar chart showing that LPS or GM-CSF has no effect on the proportion of microglia showing particle uptake after incubation with 5 or 20 μg/mL MNPs for 1 h (n = 3 cultures). **(B)** Stacked bar chart showing that LPS or GM-CSF has no effect on the extent of particle accumulation in labelled microglia. **(C)** Bar chart showing that LPS or GM-CSF has no effect on the proportion of labelled microglia with perinuclear accumulations of particles. **(D)** Stacked bar chart showing that incubation with 20 μg/mL MNPs in basal medium for 24 h has no effect on microglial morphology (n = 3 cultures).

In the absence of exogenous stimulators, the majority of OX-42 positive cells exhibited an elongated or rod-like appearance. Treatment of cultures with 100 ng/mL LPS for 24 h resulted in a marked increase in the proportion of OX-42 positive cells with rounded or amoeboid morphologies, consistent with activation. Marked changes in cell morphology were not observed in cultures pre-treated with 25 ng/mL GM-CSF for 24 h. The uptake of MNPs by microglia was not affected by pre-treatment of cultures with LPS or GM-CSF, as judged from the proportion of labelled OX-42 positive cells (Figure 4A), the extent of particle accumulation (Figure 4B) and the proportion of labelled cells displaying particles with a perinuclear localization (Figure 4C). Additionally, incubation of cells in basal medium with 20 μg/mL MNPs for 24 h had no effect on microglial morphology (Figure 4D).

### 2.4. MNP Toxicity in Microglial Cultures

**Figure 5.**
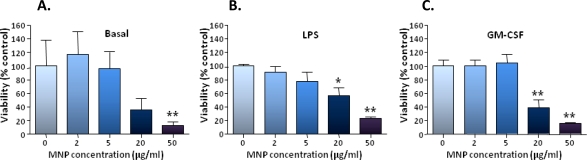
Toxicity of MNPs for microglia. Cells were incubated with indicated MNP concentrations for 24 h, then without MNPs for a further 72 h before performing a MTS assay. **(A)** Bar chart of viability data for cells cultured in basal medium alone. **(B)** Bar chart of viability data for cells cultured in medium supplemented with LPS throughout. **(C)** Bar chart of viability data for cells cultured in medium supplemented with GM-CSF throughout. *P < 0.05, **P < 0.01 and ***P < 0.001 *versus* appropriate no particle control (one-way ANOVA and Dunnett’s MCT; n = 5 cultures).

The effect of particle exposure for 24 h on cell viability was determined at 3 days post-particle incubation using a MTS assay. In the absence of an exogenous stimulator, cultures which had been pre-incubated with 50 μg/mL MNPs showed a clear-cut decrease in cell viability (mean value was 13% control value); a similar (though not statistically significant) trend was observed for cultures pre-incubated with 20 μg/mL MNPs (36% control value; Figure 5A). For cultures which had been treated with either GM-CSF or LPS, clear-cut decreases in cell viability were observed for both 20 and 50 μg/mL MNP concentrations; respective values were: 56% and 23% control values for cultures treated with LPS (Figure 5B); and 39% and 16% control values for cultures treated with GM-CSF (Figure 5C).

## 3. Discussion

The cellular uptake and toxicity of fluorescent MNPs were investigated in cultures of microglia derived from rat cerebral cortex with and without experimental activation of cultures. Experiments using cell lines have provided valuable information on the cellular dynamics of nanoparticle uptake but we consider that the use of freshly isolated cultured cells can overcome many of the drawbacks that are inherent to cell lines (contamination, aneuploidy, the need for rigorous karyotyping to assess cell identity and abnormal cell physiology secondary to transformation). We have used relatively large (0.20–0.39 μm) particles here which, in the context of biomolecule delivery, can permit more ‘loading’ of biomolecules per particle and functionalisation with multiple DNA constructs. For magnetic targeting applications, external magnetic fields would influence the movement of particles with larger dimensions (and higher iron content) to a greater extent than those with smaller dimensions. Additionally, widely used commercial, transfection grade MNPs currently employed in *in vitro* applications are of a similar size to those used in this study, which was a further consideration influencing our choice of particle. Finally, we recently reported focal uptake of these particles (following intravenous injection in a rat model of spinal cord transection) in areas of BBB disruption containing inflammatory infiltrates [21]; however, this study did not carry out a detailed characterization of cell specific particle uptake in foci of injury.

Our findings demonstrate that rat microglia avidly ingest MNPs following simple incubation of particles with cells. The primary cellular mechanism of MNP uptake is believed to be endocytosis [24]; microglial cells possess well developed endocytotic machinery and our findings on the temperature dependence of particle uptake are consistent with this being an activity dependant process. Particle uptake showed time and concentration dependence with 60–100% of cells showing some degree of labeling at 1 h after particle incubation; the extent of particle accumulation within cells also showed a marked time and concentration dependence. These results are broadly in agreement with published data demonstrating robust uptake of a range of MNPs in cultures of microglial cells/cell lines [9,12,17]. However, microglia exhibit limited or no uptake of certain classes of MNPs [17]; such differences in microglial uptake rates appear related to the physiochemical properties of particles, as reported for other cell types.

We additionally observed a prominent time-dependent “trafficking” of particles through the cytoplasm. Between 60–100% of microglia showed some degree of perinuclear localisation of particles by 1 h, suggesting that efficient intracellular transport mechanisms were being utilized; this localization was more pronounced at later time points with all cells showing perinuclear particle distribution by 24 h. Rapid trafficking of nanoparticles resulting in perinuclear accumulation has been reported previously for other cell types [22,23] and is attributed to a directed active transport process mediated by molecular motor proteins such as dyneins and kinesins [22,24]; these are nuclear transport mechanisms similar to those used by viral vectors such as adenoviruses [24].

Although chronic administration of aluminium oxide nanoparticles to rats has recently been reported to result in microglial activation in the brain [25], short-term exposure to MNPs in our experiments did not result in activation of microglia in culture, as judged by morphological criteria. However, treatment of microglial cells with LPS induced marked morphological changes characteristic of activated microglia, and both LPS and GM-CSF appeared mitogenic in MTS assays but neither treatment appeared to alter the time course or extent of particle accumulation. In contrast, others have reported that LPS treatment of microglial cell lines can induce increased uptake of MNPs [17]. However, this effect was particle-specific (observed for Endorem but not Sinerem MNPs), and was only observed when LPS and Endorem were applied simultaneously to cells [17]. Crucially, pre-activation of cells with LPS for 24 h prior to MNP addition had no effect on uptake [17], suggesting that LPS-mediated stimulation of MNP uptake is transient, most likely associated with an early step in the activation process. Taking these findings together, we can conclude that proliferating or activated microglia in culture do not exhibit increased uptake activity for a range of MNPs of different sizes and formulations.

The robust uptake of particles by microglia was found to be associated with significant toxicity at the higher particle concentrations used; nanoparticle toxicity for microglia/microglial cell lines has been noted by other workers [9,26]. We consider that the toxic effects observed in our study are likely to be related to the extent of particle uptake; however further studies are required to assess parameters such as oxidative stress and gene expression changes in microglia, following particle uptake.

We consider the observations from this study to be significant for the following reasons. First, they demonstrate that *all* microglia in culture have the capacity to take up MNPs and that the extent of particle loading of microglia (depending on the downstream applications such as transfection or imaging) can be altered by simply manipulating the initial particle incubation conditions. Second, the rapid and efficient perinuclear accumulation of MNPs in a large proportion of microglia would suggest that MNPs could act as highly effective vectors for gene delivery to microglial cells. Interestingly, however, in preliminary experiments, we have not found this to be the case. Using a range of transfection-grade MNPs and ‘magnetofection’ strategies that have been successfully applied by ourselves to astrocytes [27], we noted robust particle uptake by microglia with perinuclear localization, but very limited resultant transfection efficacy (<1%) (data not shown). It is not clear at present, what intracellular mechanisms account for the limited transfection observed. However, given the critical roles played by microglia in a range of neuropathological conditions (and the considerable benefits offered by MNP based vector systems in the context of neural regeneration), these observations suggest a clear need to (i) identify intracellular barriers to MNP-mediated transfection in microglia and; (ii) establish potential correlations between the modes of MNP uptake, intracellular MNP trafficking and their transfection capabilities, in order to develop high efficiency vectors.

Third, the robust dynamics of MNP uptake by microglial cells raise important questions in relation to particle uptake by other neural cells, in the context of neural tissue arrays involving complex interactions between neurons and glial cells. We have consistently observed that the rapidity and efficiency of MNP uptake by microglial cells results in extremely limited particle uptake by cells such as OPCs that are present in the vicinity of microglial cells. By contrast, isolated, purified populations of OPCs show significant uptake in the absence of microglia. Likewise, ultrasmall superparamagnetic iron oxide particles have been reported to be taken up almost exclusively by microglia in co-cultures of microglia and astrocytes despite demonstrable uptake of particles in pure cultures of astrocytes [9]; similar findings were reported in the same study for co-cultures of microglia plus glioma cells *versus* glioma cells alone [9]. Similarly, *post-mortem* studies of glioblastoma patients who received thermotherapy with MNPs have shown that particles are predominantly localized within macrophage-like cells, even though human neural tumour cells have the capacity to take up the particles *in vitro* [28]. Such findings suggest that *in vitro* studies reporting the dynamics of MNP uptake using isolated purified neural cell populations must be interpreted with caution, as such dynamics may be considerably altered/limited in the presence of microglia, whose nanoparticle uptake properties are comparatively more efficient. However, few studies have systematically assessed comparative nanoparticle uptake by the major neural cell populations in sites of CNS pathology. Such robust microglial uptake of MNPs is clearly of benefit when considering imaging applications to delineate sites of neural pathology (involving microglial activation). On the other hand, when considering direct MNP mediated biomolecule delivery (including gene transfer) to mixed neural cell populations containing microglia, we can predict that the rapid and extensive MNP uptake by endogenous microglia could represent a significant ‘extracellular’ barrier to particle uptake by other neural cell subpopulations. We therefore suggest that strategies aimed at developing efficient MNP vectors (for direct gene transfer to neural injury sites *in vivo*) will need to seriously consider the ‘comparative dynamics’ of particle uptake by individual neural cell types, taking into account the relative densities at which these cell types are present in the normal and injured CNS parenchyma.

Finally, a further point to consider is that immunosuppressive or immunomodulatory therapies are currently widely used in the treatment of neural injury and a range of neurodegenerative conditions. It is not clear at present, what secondary effect such therapies may have on the dynamics of particle uptake by microglia and other neural cells *in vivo*. Experimental studies using immunosuppresive drugs such as minocycline and corticosteroids, as well as methods to deplete microglial populations (such as those using clodronate liposomes) are required to address this issue.

## 4. Conclusions

Microglial cells showed robust uptake of MNPs which is time and concentration dependant; these parameters were not altered following experimental activation of the cells. Although particles showed a prominent time dependant perinuclear targeting, we argue that this may not necessarily result in efficient microglial transfection; improved particle design may be required to achieve the latter. We also argue that the highly efficient uptake dynamics of particles by microglia may limit uptake by other neural cell types which could have significant implications when considering MNP-mediated gene delivery strategies to three dimensional tissue arrays containing mixed populations of neural cells, with heterogeneous uptake dynamics.

## 5. Experimental Section

### 5.1. Cell Culture

Cerebral cortices were dissected from Sprague-Dawley rats at postnatal days 1–3 and tissue was minced after removal of meninges. Cells were mechanically dissociated in D-10 medium (Dulbecco’s modified Eagle’s medium [DMEM] supplemented with 2 mM glutaMAX-I, 1 mM sodium pyruvate, 50 U/mL penicillin, 50 μg/mL streptomycin and 10% fetal bovine serum) then sequentially filtered through 70 and 40 μm cell strainers. Dissociated cells (1.5 × 10^7^ cells) were seeded into T-75 flasks (pre-coated with poly-D-lysine) and cultured in a humidified incubator (5% CO_2_/95% air) at 37 °C for 7–10 days with periodic feeding to establish mixed glial cell cultures [29]. Microglia were obtained by mechanical shaking of mixed glial cultures on a rotary shaker in a dry incubator (37 °C; 100% air) at 200 rpm for 2 h and harvested from the medium by centrifugation. Microglia were resuspended in fresh D-10 medium and plated into poly-d-lysine coated 8-well chamber slides (6.4 × 10^4^ cells/well) or 96-well plates (2.8 × 10^4^ cells/well). In separate experiments, further shaking of mixed glial cultures for 24 h was used to generate enriched OPC preparations. Harvested OPCs were resuspended in OPC medium (DMEM supplemented with 2 mM glutaMAX-I, 1 mM sodium pyruvate, 50 U/mL penicillin, 50 μg/mL streptomycin, 0.1% bovine serum albumin, 50 μg/mL apo-transferrin, 5 μg/mL insulin, 30 nM sodium selenite, 10 nM d-biotin, 10 nM hydrocortisone, 10 ng/mL basic fibroblast growth factor and 10 ng/mL platelet-derived growth factor AA) and plated into polyornithine/laminin coated 8-well chamber slides (3.2 × 10^4^ cells/well). After plating, microglia and OPCs were allowed to attach to the substratum for 1 h, then medium was replaced with fresh medium. Cells were cultured for at least 24 h after harvesting from mixed glial cultures, before exposure to MNPs, and were not passaged further. All animal experiments were conducted in strict accordance with UK Home Office guidelines.

### 5.2. Uptake Experiments

Paramagnetic, carboxyl-modified SPHERO Nile Red fluorescent magnetic particles (0.20–0.39 μm diameter, iron content 15–20%), from Spherotech Inc. (Lake Forest, Illinois, USA), were employed in this study. The particles comprise a stained polystyrene core which is coated with a magnetite layer then overcoated with a functionalized monomer. Experiments were conducted at 37 °C in 5% CO_2_/95% air, unless otherwise specified. To determine the time course and concentration-dependence of microglial uptake, cells were incubated with 2–50 μg/mL particles in fresh D-10 medium for 1 – 24 h. To determine the effect of microglial stimulators on MNP uptake, cells were pre-treated with D-10 medium alone (controls) or D-10 medium supplemented with either lipopolysaccharide (from *E. coli* 0111:B4; 100 ng/mL) or recombinant rat GM-CSF (25 ng/mL; PeproTech, London, UK) for 24 h, then this was replaced with the same medium containing 5 or 20 μg/mL particles, and cells were incubated for a further 1 h. At the concentrations used here, GM-CSF is mitogenic for microglia but does not cause activation [19,20], whereas LPS causes microglial proliferation and activation [18]. To determine the temperature-dependence of uptake, microglia were incubated with 20 μg/mL MNPs in D-10 medium buffered with 25 mM HEPES pH 7.4 for 1 h at 4 or 37 °C (in 100% air). In all experiments, to terminate incubations, cells were rapidly washed three times with phosphate-buffered saline (PBS), then fixed with 4% (w/v) paraformaldehyde (in PBS) for 20 min. Fixed cells were washed three times with PBS. Incubation of OPC cultures with MNPs was as described for microglia except OPC medium was used.

### 5.3. Immunocytochemistry and Histochemistry

To identify microglia, fixed cells were immunostained with a primary antibody to CD11b (OX-42; Serotec, UK). Cells were first incubated with 1% Triton-X100 in PBS for 30 min at room temperature (RT) then blocked with 5% normal donkey serum in PBS for 30 min at RT. Cells were then incubated overnight at 4 °C with OX-42 (1:500 in blocker). After washing three times with PBS, a further incubation with blocker (RT for 30 min) was performed, then cells were incubated with FITC-labelled donkey anti-mouse IgG (1:200 in blocker) secondary antibody (Jackson ImmunoResearch Laboratoratories Inc., West Grove, PA, USA) for 2 h at RT. After washing three times with PBS, cells were mounted with DAPI to visualize nuclei. To identify OPCs, a similar procedure was used except the initial incubation with Triton-X100 was omitted, the blocker was 5% normal goat serum in PBS, primary antibody was anti-A2B5 (1:200 in blocker; Sigma-Aldrich, Poole, Dorset, UK) and secondary antibody was FITC-labelled goat anti-mouse IgM (1:200 in blocker; Jackson ImmunoResearch Laboratoratories Inc., West Grove, PA, USA). For the Perl’s Prussian Blue staining method to visualize intracellular iron, fixed microglia were incubated with 2% potassium ferricyanide in 2% HCl for 30 min, washed three times with distilled water and mounted without DAPI.

### 5.4. Microscopy and Image Analysis

Fluorescence, phase contrast and light microscopy were performed using an Axio Scope A1 microscope equipped with an Axio Cam ICc1 camera (Carl Zeiss MicroImaging GmbH, Goettingen, Germany). Purity of microglial cultures was assessed from merged images of OX-42 and DAPI stained cells; at least 100 DAPI-stained nuclei were scored for coincident OX-42 staining. Purity of OPC cultures was assessed from merged images of A2B5 and DAPI staining. Particle uptake by microglia was assessed from triple merges (X400 magnification) of DAPI, Nile Red and OX-42 fluorescent images, and the proportion of microglia (OX-42 positive cells) demonstrating particle uptake (termed % labelled cells) was calculated. We have used a semi – quantitative method to estimate the extent of particle accumulation, labelled microglia were categorized based on the area of the cell occupied by the accumulated nanoparticles relative to the area of the cell nucleus; categories were: low (≤10%), moderate (11–50%) and high (≥51%). Labelled microglia were also classified with respect to the subcellular localization of nanoparticles; the proportion of labelled cells with a perinuclear particle localization either exclusively or combined with a cytoplasmic particle distribution is reported here. To assess co-localization of MNPs and iron-containing material, images of cells (after Perl’s staining) taken by standard light microscopy and by fluorescence microscopy (Nile Red filter) were merged. Microglial morphology was assessed using phase-contrast microscopy; cells were classified as displaying: (i) a rounded or amoeboid morphology; (ii) an elongated or rod-like morphology, usually unipolar or bipolar (‘rod-like’) and; (iii) a more highly ramified appearance (‘ramified’). For all calculations, a minimum of 100 OX-42-positive cells per treatment were evaluated. To determine if cell-associated particles had been internalized rather than simply adsorbed onto the cell surface, a series of Z-stacks of 50 cells (labelled with DAPI, OX-42 and MNPs) at 1 μm depths were manually acquired using a Nikon Eclipse 80i microscope, equipped with filter blocks UV-2E/C (DAPI), B-2E/C (FITC) and G-2E/C (TRITC; for Nile Red). Stacks were merged, then viewed after combination and as a montage of tiled images; image manipulations were performed using Nikon NIS elements (version 3.00).

### 5.5. SEM

To assess particle size and shape, Spherofluor particles were placed in pure water, allowed to air dry onto aluminium stubs and visualised uncoated using a high resolution field emission SEM (Hitachi S4500) operated at an accelerating voltage of 5 kV.

### 5.6. Cell Viability Assays

Microglia were incubated with D-10 medium alone or D-10 medium supplemented with either GM-CSF (25 ng/mL) or LPS (100 ng/mL) for 24 h then with the same medium further supplemented with 0–50 μg/mL MNPs for 24 h in a final volume of 0.2 mL medium per well. Medium was replaced with fresh D-10 medium (plus activator, as appropriate) and cells were incubated for a further 72 h. Then 20 μL MTS reagent (CellTiter 96 AQueous One Solution Cell Proliferation Assay; Promega, Southampton, UK) was added, plates were incubated for a further 3 h at 37 °C, and the absorbance at 490 nm (A490nm) was determined. Values were corrected for the appropriate medium blank reading; medium blanks comprised wells minus cells which were treated in an identical manner to wells plus cells.

### 5.7. Statistical Analysis

Data are expressed as mean ± SEM. To assess the effects of MNP concentration and time of exposure on proportion of cells showing particle uptake, extent of particle accumulation and degree of perinuclear localisation, data were analyzed by a two-way ANOVA followed by Bonferroni’s post-tests. For temperature-dependence of uptake, data were analyzed by Student’s *t*-test (two-tailed). One-way ANOVA, with Dunnett’s MCT was used to analyze data for the cytotoxicity experiments.
